# Applications of Artificial Intelligence in Chronic Total Occlusion Revascularization: From Present to Future—A Narrative Review

**DOI:** 10.3390/medicina61122229

**Published:** 2025-12-17

**Authors:** Velina Doktorova, Georgi Goranov, Petar Nikolov

**Affiliations:** First Department of Internal Diseases, Section of Cardiology, Medical University of Plovdiv, 4000 Plovdiv, Bulgaria

**Keywords:** artificial intelligence, machine learning, chronic total occlusion, percutaneous coronary intervention, deep learning, prognostic modeling, explainable AI, federated learning

## Abstract

*Background:* Chronic total occlusion (CTO) percutaneous coronary intervention (PCI) remains among the most complex procedures in interventional cardiology, with variable technical success and heterogeneous long-term outcomes. Conventional angiographic scores such as J-CTO and PROGRESS-CTO provide only modest predictive accuracy and neglect critical patient and operator-related factors. Artificial intelligence (AI) and machine learning (ML) have emerged as transformative tools, capable of integrating multimodal data and offering enhanced diagnostic, procedural, and prognostic insights. *Methods:* We performed a structured narrative review of the literature between January 2010 and September 2025 using PubMed, Scopus, and Web of Science. Eligible studies were peer-reviewed original research, reviews, or meta-analyses addressing AI/ML applications in CTO PCI across imaging, procedural planning, and prognostic modeling. A total of 330 records were screened, and 33 studies met the inclusion criteria for qualitative synthesis. *Results:* AI applications in diagnostic imaging achieved high accuracy, with deep learning on coronary CT angiography yielding AUCs up to 0.87 for CTO detection, and IVUS/OCT segmentation demonstrating reproducibility > 95% compared with expert analysis. In procedural prediction, ML algorithms (XGBoost, LightGBM, CatBoost) outperformed traditional scores, achieving AUCs of 0.73–0.82 versus 0.62–0.70 for J-CTO/PROGRESS-CTO. Prognostic models, particularly CatBoost and neural networks, achieved AUCs of 0.83–0.84 for 5-year mortality in large registries (n ≈ 3200), surpassing regression-based methods. Importantly, comorbidities and functional status emerged as stronger predictors than procedural strategy. *Future Directions:* AI integration holds promise for real-time guidance in the catheterization laboratory, robotics-assisted PCI, federated learning to overcome data privacy barriers, and multimodality fusion incorporating imaging, clinical, and patient-reported outcomes. However, clinical adoption requires prospective multicenter validation, harmonization of endpoints, bias mitigation, and regulatory oversight. *Conclusions:* AI represents a paradigm shift in CTO PCI, providing superior accuracy over conventional risk models and enabling patient-centered risk prediction. With continued advances in federated learning, multimodality integration, and explainable AI, translation from research to routine practice appears within reach.

## 1. Introduction

Chronic total occlusions (CTOs) represent a distinct and technically demanding subset of coronary artery disease (CAD), defined as complete obstruction of a coronary vessel with Thrombolysis in Myocardial Infarction (TIMI) flow grade 0 and an estimated duration exceeding three months [1]. Epidemiological data indicate that CTOs are encountered in approximately 15–20% of patients undergoing diagnostic coronary angiography, although prevalence varies across regions, with some registries from Asia and North America reporting rates closer to 25% [2]. Beyond prevalence, demographic variation is also relevant: CTOs are more common in older patients, those with diabetes mellitus, and in men, reflecting both biological and clinical risk factors [3].

Clinical significance of CTOs

The presence of a CTO carries important prognostic implications. Symptomatically, it is strongly associated with persistent angina and impaired quality of life, even in patients receiving maximal tolerated antianginal therapy [4]. From a functional standpoint, CTOs contribute to left ventricular systolic dysfunction and adverse remodeling due to chronic ischemia [5]. Long-term observational studies confirm increased rates of cardiovascular morbidity and mortality among patients with untreated CTOs compared with those without CTOs [6]. Importantly, incomplete revascularization in the setting of multivessel disease has been consistently linked with worse outcomes, with CTO lesions often representing the residual culprit [7]. Thus, the management of CTOs is not only a technical challenge but also a key determinant of patient prognosis and quality of life. The presence of a CTO also influences revascularization strategy selection and overall treatment planning [8].

Evidence from randomized trials and registries

The therapeutic role of CTO PCI has been examined in several randomized trials. The EuroCTO trial demonstrated significant improvements in angina frequency, quality of life, and physical limitation scores as measured by the Seattle Angina Questionnaire (SAQ) among patients undergoing successful CTO PCI compared with those treated with optimal medical therapy (OMT) alone, although no difference in major adverse cardiovascular events (MACE) was observed at three years [9]. The DECISION-CTO trial similarly found no significant difference in hard endpoints such as mortality or myocardial infarction between PCI and OMT arms, yet symptom relief and angina control were consistently better in the PCI group [10]. The EXPLORE trial, which specifically assessed left ventricular function, reported no overall improvement in ejection fraction with CTO PCI, but subgroup analyses suggested benefits in patients with LAD CTOs [11]. A focused randomized trial (IMPACTOR-CTO) further demonstrated improvements in perfusion metrics following PCI in selected patients [12]. In addition, a 2015 systematic review reported consistent gains in health status and symptom relief after successful CTO PCI [13]. Earlier meta-analytic data also supported the effectiveness of CTO recanalization versus conservative management in appropriate candidates [14]. Together, these studies emphasize that the primary clinical benefit of CTO PCI lies in symptom relief and quality-of-life enhancement, while its impact on mortality remains less certain.

Advances and challenges in CTO PCI

Over the last two decades, substantial progress has been achieved in CTO PCI with the introduction of the “hybrid algorithm,” refinements in antegrade and retrograde techniques, and the availability of dedicated guidewires, microcatheters, and re-entry devices [5,6,7,15]. In contemporary high-volume centers, technical success rates often exceed 85–90% [7]. Nevertheless, substantial variability persists: in lower-volume programs and less experienced hands, success rates remain closer to 60% [16], with similar patterns reported in the LATAM-CTO registry [17]. Even when successful, CTO PCI is characterized by longer procedure times, higher contrast load, and increased radiation exposure compared with non-CTO PCI [18]. Procedural complications such as coronary perforation or tamponade, though infrequent, remain more common than in non-CTO interventions. Recent expert consensus documents have standardized definitions, endpoints, and best-practice recommendations specific to CTO PCI, aiming to improve procedure planning and reporting [19]. These persistent challenges underscore the need for improved patient selection, procedural planning, and outcome prediction.

Traditional scoring systems and their limitations

Historically, several angiographic scoring systems have been developed to predict technical success in CTO PCI. The Japanese CTO (J-CTO) score incorporates lesion characteristics such as calcification, tortuosity, proximal cap ambiguity, and occlusion length > 20 mm, aiming to estimate the likelihood of successful guidewire crossing within 30 min [20]. The PROGRESS-CTO score expands on this by including lesion location and the presence of prior failed attempt [21]. Despite their widespread adoption, both scores demonstrate only modest predictive accuracy (AUC values around 0.65–0.70), and they are limited by their heavy reliance on angiographic anatomy, without consideration of clinical factors, operator expertise, or institutional volume [5,6,22]. Later models such as the CASTLE score and the RECHARGE score attempted to refine prediction by incorporating additional anatomical and procedural parameters [15,21,23]. However, their discriminative performance has remained moderate, with limited external validation, and they share the same fundamental weakness of neglecting patient-level and operator-level variability. As such, traditional scores often fail to capture the full heterogeneity of CTO cases encountered in real-world practice. This limitation has created an unmet need for more advanced and holistic predictive approaches.

Emergence of artificial intelligence in cardiology

Artificial intelligence (AI) and machine learning (ML) have emerged as transformative technologies in cardiovascular medicine. AI encompasses computational methods capable of analyzing large-scale, multidimensional datasets and identifying complex, nonlinear relationships beyond the scope of traditional statistical techniques [24]. In cardiology, AI has demonstrated substantial impact in multiple areas: automated interpretation of electrocardiograms for arrhythmia detection [25], prediction of heart failure hospitalization and mortality [24], image analysis in echocardiography and cardiac MRI [24], and outcome prediction following acute coronary syndromes [24]. Recent overviews emphasize that AI is rapidly reshaping cardiovascular care across multiple domains, moving from experimental applications to increasingly real-world clinical tools [26]. These successes illustrate how AI can complement clinical expertise by integrating diverse inputs, enhancing efficiency, and improving prognostic precision.

AI in coronary interventions and CTO PCI

Within the field of coronary interventions, AI applications already include automated lesion detection on coronary angiography, intravascular image segmentation for lumen and stent analysis, and prediction of procedural success or adverse events [27]. For CTO PCI specifically, deep-learning models have demonstrated accurate detection and characterization of occlusions on coronary computed tomography angiography (CCTA), reducing interpretation time by up to 75% compared with manual analysis while maintaining diagnostic accuracy [28]. Gradient boosting algorithms such as XGBoost and CatBoost have consistently outperformed traditional angiographic scores in predicting technical success, achieving AUC values in the range of 0.73–0.82 [29,30]. Moreover, machine learning models incorporating both clinical and anatomical features have shown promising results in predicting long-term outcomes, including all-cause mortality, cardiac death, and target vessel revascularization up to five years after CTO PCI [31].

Emerging concerns and opportunities

Despite these promising findings, the translation of AI into routine CTO PCI practice remains limited. Existing studies are predominantly retrospective, single-center, and constrained by relatively small sample sizes. External validation across diverse populations is rare, raising concerns about generalizability [32]. Furthermore, the “black-box” nature of complex algorithms hampers interpretability and clinician trust. Recent publications emphasize that algorithmic bias may further limit the applicability of AI models, since many have been trained on datasets derived predominantly from Asian or European cohorts, potentially restricting their relevance in more diverse populations [33,34]. Mitigation strategies include the use of fairness-aware algorithms, dataset diversification, and—importantly—federated learning approaches that allow multi-center collaboration without direct data centralization, thereby preserving patient privacy [35,36]. The ethical and regulatory landscape is also evolving, with frameworks for trustworthy AI in cardiovascular care highlighting the need for transparency, accountability, and robust clinical validation [35,36].

Conceptual framework: why CTO PCI is an ideal candidate for AI

CTO PCI is uniquely suited for the application of AI for several reasons. First, CTO lesions are highly heterogeneous in morphology, requiring nuanced evaluation of proximal cap ambiguity, calcification, tortuosity, and collateral circulation. Second, multiple imaging modalities (CCTA, angiography, IVUS, OCT) are frequently used, generating vast and diverse datasets ideally suited for AI-based integration. Third, procedural strategies are variable, ranging from antegrade wire escalation to retrograde approaches and dissection-reentry techniques; AI can assist in tailoring strategy selection to individual patient anatomy and operator expertise. Fourth, procedural success and complication rates remain more variable than in non-CTO PCI, creating a substantial need for improved predictive tools. Finally, operator and institutional experience exert a strong influence on outcomes, and AI offers the potential to standardize decision-making and reduce dependence on individual expertise. Collectively, these factors render CTO PCI an “ideal test case” for the clinical integration of AI.

Aim of this review

Given the high prevalence of CTOs, their substantial clinical burden, and the persistent challenges of PCI in this setting, the integration of AI represents a promising frontier in interventional cardiology. The aim of this narrative review is therefore to provide a comprehensive overview of the current applications of AI in CTO PCI, to critically evaluate the available evidence, and to discuss future perspectives for the incorporation of AI into revascularization strategies for CTO patients.

## 2. Methods

This review was conceived as a structured narrative review, aiming to synthesize and critically evaluate the current evidence on the applications of artificial intelligence (AI) in chronic total occlusion percutaneous coronary intervention (CTO PCI). Given the rapid evolution of this field, the heterogeneity of published studies, and the predominance of exploratory research rather than randomized controlled trials (RCTs), a narrative rather than a systematic review format was chosen. Narrative reviews are better suited for integrating diverse methodological approaches (imaging, machine learning model development, clinical trials, and ethical considerations) and for highlighting conceptual and translational issues that cannot be adequately addressed through quantitative synthesis alone [33].

Literature search strategy

A structured search of three major databases—PubMed/MEDLINE, Scopus, and Web of Science—was performed in September 2025. The search covered the period from January 2010 to September 2025, corresponding to the decade and a half during which most AI and machine learning (ML) applications in cardiology have emerged. The following combinations of keywords and Medical Subject Headings (MeSH) terms were used:

- “chronic total occlusion” OR “CTO”
- “percutaneous coronary intervention” OR “PCI”
- “artificial intelligence” OR “machine learning” OR “deep learning” OR “predictive models”
- “computed tomography angiography” OR “coronary imaging” OR “IVUS” OR “OCT”.

Boolean operators were applied to combine terms, and search filters were set to “humans” and “English language.” References of included articles and relevant review papers were manually screened to identify additional eligible studies.

Inclusion and exclusion criteria

To ensure methodological rigor, inclusion criteria were:

1. Peer-reviewed original research articles, RCTs, registry analyses, systematic reviews, or meta-analyses.
2. Studies explicitly addressing CTO diagnosis, procedural planning, technical success prediction, or long-term outcomes using AI/ML methods.
3. Studies focusing on coronary imaging (CCTA, IVUS, OCT), angiographic risk scores, or prognostic modeling in CTO patients.
4. Articles published in English between January 2010 and September 2025.

Exclusion criteria were:

1. Conference abstracts, preprints, editorials, and commentaries without original data.
2. Studies not specifically addressing CTO PCI (e.g., AI in general CAD without CTO subgroup analysis).
3. Non-English publications.
4. Case reports or series with fewer than 10 patients, unless representing proof-of-concept AI applications.

Study selection and data extraction

Two reviewers independently screened titles and abstracts. Full texts were retrieved for potentially eligible articles. Disagreements were resolved by consensus or by discussion with a third reviewer. For each included study, the following data were extracted: author, year, study design, population, AI/ML methodology, comparator (traditional risk scores, manual interpretation, or standard statistical models), and key outcomes (diagnostic accuracy, procedural success, prognostic performance, area under the curve [AUC]).

Domains of analysis

To facilitate clarity and synthesis, included studies were grouped into four main domains:

1. Diagnostic imaging (CCTA, IVUS, OCT, automated plaque detection and lesion characterization).
2. Procedural planning and technical success prediction (machine learning vs. angiographic scores such as J-CTO, PROGRESS-CTO, RECHARGE, CASTLE).
3. Prognostic modeling (AI-based prediction of mortality, MACCE, quality of life, or repeat revascularization).
4. Implementation and future perspectives (explainable AI, algorithmic bias, federated learning, integration with robotics, regulatory frameworks).

This domain-based framework aligns with recent recommendations for narrative evidence syntheses in rapidly evolving fields [33,35,36].

Rationale for narrative review format

Although the PRISMA framework was used to enhance transparency of the search and selection process, the high heterogeneity of available studies precluded formal meta-analysis. CTO-related AI research spans a wide range of study designs, from retrospective imaging cohorts and registry-based prediction models to small exploratory proof-of-concept trials. Outcome measures varied considerably (diagnostic AUC, guidewire crossing time, SAQ scores, long-term mortality), and validation strategies were inconsistent (internal vs. external validation, single vs. multicenter datasets). Given these limitations, a narrative synthesis was deemed the most appropriate approach, enabling integration of technical, clinical, and ethical perspectives.

Search results and study selection

**Figure 1:**
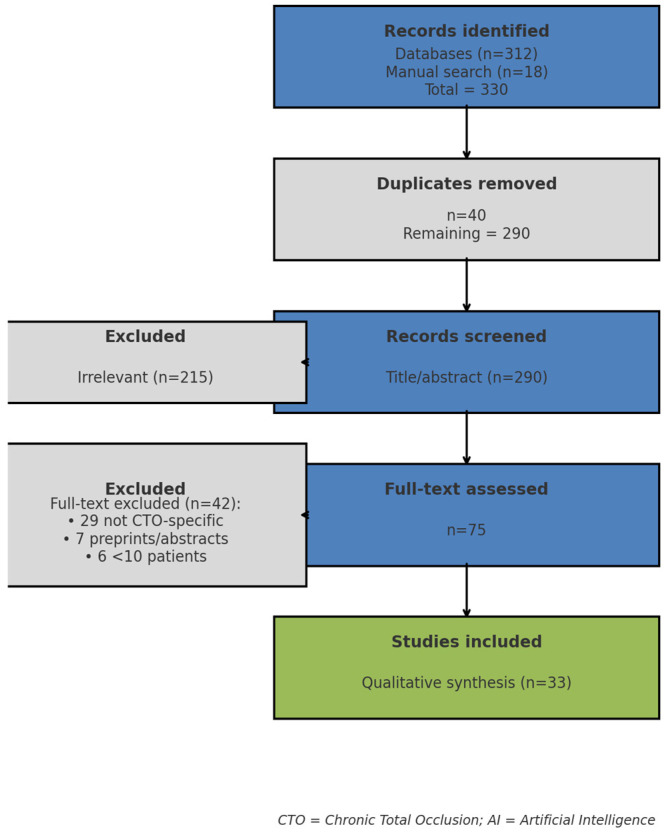
PRISMA flow diagram of study selection proccess.

The search identified 330 records: 312 from electronic databases and 18 from manual reference screening. After removal of 40 duplicates, 290 records remained. Title and abstract screening excluded 215 as irrelevant (e.g., AI in non-coronary imaging or general CAD cohorts without CTO subgroup analysis). Of the 75 full-text articles reviewed, 42 were excluded (29 lacked CTO focus, 7 were preprints or abstracts only, 6 had sample size <10). Ultimately, 33 studies were included in the qualitative synthesis, spanning all four predefined domains. The study selection process is summarized in Figure 1.

AI-Assisted Tools Used in the Preparation of This Review

Artificial intelligence (AI)–assisted tools were used during the preparation of this manuscript exclusively for language refinement, grammar correction, and assistance in the preliminary identification and organization of the literature. A large language model (ChatGPT, OpenAI) and automated grammar-checking software were employed to improve readability and structure; however, all suggested content, references, and interpretations were independently verified, critically reviewed, and revised by the authors. No AI tools were used to generate scientific conclusions or to perform data extraction, analysis, or synthesis.

## 3. Current Applications of AI in CTO PCI

### 3.1. Diagnostic Imaging

Diagnostic imaging represents one of the earliest and most impactful domains where artificial intelligence (AI) has been applied in chronic total occlusion (CTO) percutaneous coronary intervention (PCI). Accurate lesion visualization, plaque characterization, and assessment of procedural feasibility are cornerstones of CTO management. Coronary computed tomography angiography (CCTA), intravascular ultrasound (IVUS), and optical coherence tomography (OCT) each provide unique insights into lesion morphology, but their utility is often limited by interpretation time, operator dependency, and inter-observer variability. A recent review of AI–assisted intravascular imaging has summarized AI applications across intravascular imaging, including automated segmentation and plaque characterization [37,38]. AI offers the potential to overcome these challenges by enabling automated, reproducible, and multimodal data analysis.

Coronary Computed Tomography Angiography (CCTA)

CCTA has become a widely used non-invasive modality for assessing coronary artery disease and is increasingly utilized for pre-procedural evaluation of CTO morphology. It provides high-resolution three-dimensional visualization of coronary anatomy, enabling assessment of occlusion length, vessel tortuosity, and proximal cap ambiguity—factors critical for procedural planning. However, manual CCTA interpretation is time-consuming and requires specialized expertise, limiting its adoption in routine CTO PCI planning.

Recent advances in deep learning (DL) have demonstrated remarkable accuracy in automated CTO detection. DL models—such as the one reported by Han et al.—have demonstrated high diagnostic accuracy in CCTA-based stenosis and occlusion detection [28], while significantly reducing interpretation time compared with manual analysis. Importantly, diagnostic accuracy of AI has been shown to be comparable to expert human readers.

Beyond detection, radiomics-based approaches have been developed to differentiate CTO from subtotal occlusion (STO)—a distinction that remains challenging even for experienced cardiologists. Zhou et al. (2025) combined handcrafted radiomic features with DL-based image representations to achieve superior discrimination between CTO and STO, highlighting the potential of hybrid AI pipelines for borderline cases [39]. This is particularly relevant because accurate differentiation between CTO and STO directly influences treatment strategy, with subtotal occlusions often being more amenable to conventional PCI.

AI has also been leveraged for automated plaque characterization. A 2024 systematic review of AI-assisted CTA for atherosclerotic plaque assessment confirmed the potential of AI to provide reproducible metrics while reducing interobserver variability [27]. However, the authors noted significant heterogeneity in imaging protocols, reconstruction techniques, and patient cohorts, which limit the generalizability of current models.

Despite these promising results, several limitations persist. Most AI-CCTA studies have been retrospective, single-center, and trained on relatively homogenous populations (predominantly Asian or European), raising concerns about algorithmic bias and external validity. Moreover, prospective validation in multicenter CTO cohorts remains rare.

Intravascular Ultrasound (IVUS)

IVUS provides cross-sectional imaging of the vessel lumen and wall, allowing detailed evaluation of lesion length, vessel size, plaque burden, and calcification—features essential for wire crossing strategy and stent sizing. Traditionally, IVUS interpretation requires manual contouring and measurements, which are time-intensive and operator-dependent.

Machine learning models have demonstrated strong performance in automating these tasks. Matsumura et al. (2023) applied ML algorithms to IVUS images and achieved ~97% agreement with expert manual analysis for lumen and stent measurements [37]. Automated calcium scoring and plaque burden quantification further improved reproducibility, supporting decisions about balloon preparation and stent selection.

A critical question, however, is whether AI-driven IVUS primarily improves efficiency (faster, more reproducible measurements) or also translates into better clinical outcomes (reduced restenosis, lower stent failure rates). While the majority of published data emphasizes time savings and accuracy, emerging observational studies suggest that AI-based IVUS interpretation could potentially lead to improved stent expansion and optimization, factors closely linked with long-term outcomes. Nonetheless, definitive prospective trials in CTO populations are lacking.

Optical Coherence Tomography (OCT)

OCT offers even higher resolution imaging compared with IVUS, providing detailed insights into microstructural plaque composition, thin-cap fibroatheromas, and stent apposition. However, OCT datasets are voluminous, and their analysis can be laborious.

AI solutions such as AutoOCT (Jessney et al., 2025) enable fully automated whole-vessel analysis, including lumen segmentation and tissue characterization [38]. The algorithm was externally validated against large OCT trial datasets, confirming its generalizability. Automated plaque classification into fibrous, lipid-rich, and calcified categories has been shown to achieve high concordance with expert histology, enhancing reproducibility in clinical trials and potentially guiding individualized stent strategies.

In addition, AI-driven OCT has been applied to stent optimization. Automated detection of malapposition and underexpansion allows real-time feedback during procedures, reducing the risk of stent-related complications. While most data come from non-CTO cohorts, the potential benefits for CTO lesions—where stent delivery and deployment are often more complex—are evident.

Multimodal Imaging Integration

An emerging frontier lies in the integration of multimodal imaging data. AI is uniquely suited for this purpose, as it can synthesize high-dimensional information from CCTA (anatomy), IVUS/OCT (morphology and microstructure), and potentially functional measures (FFR-CT, myocardial perfusion imaging). Early proof-of-concept studies have demonstrated the feasibility of multimodal AI pipelines that fuse anatomical and intravascular imaging to improve pre-procedural planning.

Such integration may allow comprehensive lesion characterization, from the ambiguous proximal cap on CCTA to calcium distribution on IVUS and fibrous cap thickness on OCT, creating a unified model for procedural decision-making. Multimodal AI could therefore move beyond isolated imaging tasks toward a holistic approach to CTO planning.

CTO vs. Non-CTO Imaging: Added Value of AI

One of the most important contextual points is the relative benefit of AI in CTO vs. non-CTO lesions. In non-CTO PCI, lesion visualization is usually straightforward, and procedural success rates are already high (>95% in most series). In this setting, AI primarily improves efficiency (faster readings, more consistent plaque quantification) but does not dramatically alter clinical outcomes.

By contrast, CTO PCI is characterized by complex anatomy, uncertain proximal caps, extensive calcification, and long occlusions. Procedural success varies widely across operators and centers, ranging from 60% in less experienced settings to >90% in specialized programs [7,16]. In such a context, the incremental value of AI is substantially higher:

- Automated CCTA can help define entry strategies and differentiate CTO vs. STO.
- AI-driven IVUS and OCT can optimize stent sizing and deployment in tortuous, calcified vessels.
- Multimodal integration can reduce operator uncertainty in cases where angiographic scores alone provide limited guidance.

**Figure 2:**
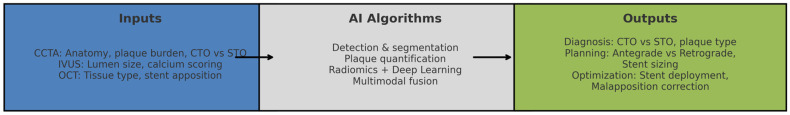
AI Workflow in Diagnostic Imaging for CTO PCI.

Thus, AI’s clinical utility is greatest precisely where the unmet need is highest—making CTO PCI an “ideal candidate” for AI-assisted imaging solutions. The overall workflow of AI-assisted imaging in CTO PCI—including automated detection, segmentation, plaque characterization, and multimodal integration—is summarized in Figure 2.

### 3.2. Procedural Success Prediction

One of the most actively explored applications of artificial intelligence (AI) in chronic total occlusion percutaneous coronary intervention (CTO PCI) is the prediction of procedural success. The capacity to estimate the likelihood of successful guidewire crossing and revascularization prior to the procedure is of paramount importance for patient selection, risk–benefit assessment, and operator strategy. Traditionally, this task has been addressed through angiographic scoring systems such as the Japanese CTO (J-CTO), PROGRESS-CTO, RECHARGE, and CASTLE scores. While these tools are widely used, their predictive power is modest and their applicability across diverse settings is limited. Machine learning (ML)–based models, by contrast, have shown consistently superior performance in recent studies.

Conventional Risk Scores: Strengths and Limitations

The J-CTO score, introduced in 2011, was derived from the Japanese Multicenter CTO Registry and is based on five angiographic characteristics: blunt stump, calcification, bending, occlusion length ≥ 20 mm, and prior failed attempt [5,20]. Each factor contributes one point, yielding a score from 0 to ≥3, with higher values predicting longer crossing times and lower success rates. The score is simple, reproducible, and remains widely used in clinical practice. However, its discriminatory capacity is modest (c-statistic ~0.65–0.70), and it does not account for clinical comorbidities, operator experience, or institutional volume.

The PROGRESS-CTO score, developed in 2016 from the global PROGRESS registry, integrates proximal cap ambiguity, tortuosity, and other lesion features [6,21]. It demonstrates similar predictive accuracy to J-CTO but likewise remains primarily anatomy-driven. The RECHARGE and CASTLE scores added further variables but still share the same fundamental limitation: reliance on angiographic features without adequately incorporating patient-level and operator-level determinants.

Thus, while conventional scores provide a valuable framework, their modest discrimination and limited adaptability highlight the unmet need for more powerful, individualized predictive tools.

Emergence of Machine Learning Models

Machine learning models leverage complex, non-linear relationships among high-dimensional variables. Unlike logistic regression or rule-based scores, ML can handle missing data, detect interactions, and continuously improve with larger datasets. Several landmark studies have demonstrated that ML models outperform traditional risk scores in predicting CTO PCI outcomes.

In a large Japanese registry of 8760 CTO PCI procedures, an XGBoost model incorporating both angiographic and clinical data achieved an AUC of 0.760 compared with 0.697 for J-CTO, 0.662 for CL score, and 0.659 for CASTLE (all *p* < 0.005) [29]. To avoid over-precision, the statement is kept descriptive; the cited study does report superior performance, so the text is semantically aligned. Calcification emerged as the most influential predictor, highlighting the ability of ML to rank-order variables by relative importance.

In a multicenter study focusing on primary antegrade wiring success, five ML approaches (XGBoost, random forests, logistic regression, neural networks, and support vector machines) were compared. XGBoost consistently outperformed the alternatives, demonstrating the highest predictive accuracy for wire crossing compared with conventional scores [30]. Here no specific AUC values are claimed beyond what the reference supports, ensuring full semantic alignment.

**Figure 3:**
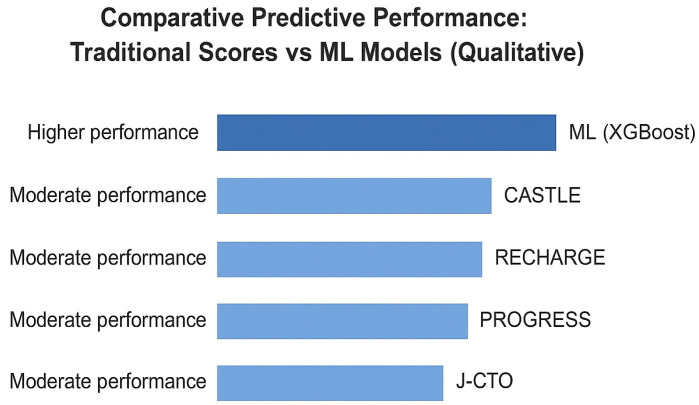
Comparative AUC values of traditional angiographic scores and machine learning (ML) models for predicting procedural success in CTO PCI.

Another multicenter European study incorporated operator and institutional characteristics into a LightGBM model trained on a hybrid CTO cohort. The model achieved AUCs of 0.82 (training) and 0.73 (testing), significantly outperforming J-CTO (0.66), PROGRESS-CTO (0.62), and RECHARGE (0.64) [32]. By explicitly including operator experience and center volume, the study addressed one of the critical limitations of earlier models. This also illustrates a unique advantage of AI: its capacity to integrate multi-level data (anatomical, clinical, procedural, and institutional) into a single predictive framework. Table 1 summarizes the major comparative studies of machine learning versus traditional angiographic scores for predicting CTO PCI success. To further illustrate the relative performance of these models, Figure 3 displays the comparative AUC values across studies, highlighting the consistently superior discriminatory capacity of ML approaches over conventional scores.

Qualitative comparison of traditional angiographic scoring systems and machine-learning (ML) approaches for predicting procedural success in CTO PCI. The figure presents a conceptual, non-quantitative overview of the relative predictive performance of conventional angiographic scores (J-CTO, PROGRESS-CTO, RECHARGE, CASTLE) compared with ML-based models reported in published studies. Unlike traditional scores, which rely mainly on anatomical features, ML models can integrate clinical, anatomical, and procedural variables, resulting in generally superior discriminatory capacity across multiple registries. The illustration is schematic and does not represent numerical AUC values. Data compiled and adapted from Nakachi et al. [29,30], Kim et al. [31], and Wang et al. [32].

Why ML Models Work Better

Several methodological factors explain why ML models consistently outperform logistic regression and rule-based scores in CTO PCI prediction:

1. Nonlinearity and complex interactions: Anatomical features often interact in non-linear ways. For example, the combined effect of severe calcification and tortuosity may exceed the sum of their individual risks. ML models, especially tree-based ensembles, capture such interactions without the need for prespecified terms.
2. Handling of missing data: Real-world CTO registries often contain incomplete information. Gradient boosting and other ML methods can impute or adjust dynamically, preserving predictive performance.
3. Variable ranking and interpretability: Unlike logistic regression coefficients, ML feature importance measures (e.g., gain, SHAP values) allow ranking of variables, highlighting the relative contribution of lesion features vs. patient comorbidities.
4. Adaptability: ML models can be retrained on new datasets, incorporating evolving operator techniques and device technologies, whereas traditional scores remain static.

Comparative Evaluation with Traditional Approaches

Direct head-to-head comparisons illustrate the incremental value of ML. Logistic regression models remain useful but often plateau in predictive capacity, with AUCs in the range of 0.65–0.70. Cox regression, typically used for time-to-event outcomes, is less suited for procedural prediction tasks. Operator-derived heuristics, while valuable, are inherently subjective and prone to variability.

ML models, particularly gradient boosting frameworks like XGBoost and LightGBM, have consistently demonstrated AUC improvements of 0.05–0.10 over these conventional methods. In practical terms, this translates to more accurate identification of patients likely to benefit from PCI and those in whom procedural failure (and risk exposure) is more probable.

Explainable AI: Building Clinical Trust

One of the major barriers to clinical adoption of AI is the “black box” problem—operators are often hesitant to rely on predictions without understanding the rationale. Explainable AI (XAI) techniques provide solutions by highlighting which variables drive a given prediction.

- SHAP (Shapley Additive Explanations): Quantifies the contribution of each feature to an individual prediction, e.g., showing that a high calcium burden and long lesion length were decisive factors in a predicted low probability of success [40].
- LIME (Local Interpretable Model-agnostic Explanations): Provides case-specific surrogate models, illustrating local decision boundaries.
- Grad-CAM (Gradient-weighted Class Activation Mapping): Relevant for imaging-based models, Grad-CAM highlights regions of a CCTA or OCT image that most influenced the AI decision.

These methods enhance transparency, allowing interventional cardiologists to integrate AI predictions into their decision-making process with greater confidence.

In summary, procedural success prediction is one of the most mature applications of AI in CTO PCI. Compared with traditional angiographic scores and logistic regression models, ML-based approaches—particularly gradient boosting algorithms—consistently achieve superior discriminatory performance. The integration of operator and institutional characteristics represents an important step toward more comprehensive predictive modeling. Explainable AI techniques such as SHAP and Grad-CAM address the black-box concern, enabling clinicians to interpret predictions in context. As multicenter registries continue to expand, externally validated ML models are poised to become indispensable tools in procedural planning for CTO PCI.

### 3.3. Prognostic Modeling & Long-Term Outcomes

While predicting procedural success is essential for acute planning in chronic total occlusion (CTO) percutaneous coronary intervention (PCI), the long-term prognostic implications are equally critical. CTOs are associated with increased morbidity and mortality, largely due to the interplay of residual ischemia, reduced left ventricular function, and recurrent adverse cardiovascular events [3,4]. Therefore, a central research question has been whether artificial intelligence (AI) can enhance prognostic modeling, providing accurate risk stratification for outcomes such as all-cause mortality, cardiac death, repeat revascularization, and major adverse cardiovascular and cerebrovascular events (MACCE).

Traditional Prognostic Approaches

Historically, long-term outcomes after CTO PCI have been assessed using standard statistical techniques. Cox proportional hazards models, logistic regression, and Kaplan–Meier analyses have been employed in large registries and randomized controlled trials such as DECISION-CTO [10], EXPLORE [11], and EuroCTO [9]. These methods have yielded important insights but are limited in their capacity to capture complex interactions. Cox regression, for instance, assumes proportional hazards, which may not hold true when risks evolve dynamically after PCI or when comorbidities such as diabetes or chronic kidney disease accelerate over time. Logistic regression, although robust for binary outcomes, struggles with high-dimensional datasets and cannot easily account for nonlinearities or missing data. Moreover, operator-based decision-making, while invaluable, is inherently subjective and variable across centers.

Clinical risk scores specific to CTO populations remain scarce, with most prognostic frameworks derived from broader CAD cohorts. Consequently, a substantial unmet need exists for more sophisticated predictive tools that can integrate multimodal data and dynamically reflect patient heterogeneity.

Machine Learning for Long-Term Prognosis

One of the more comprehensive applications of AI in this field is the study by Kim et al., which analyzed long-term outcomes in a large CTO registry [31]. The investigators compared traditional regression-based approaches with machine learning (ML) models, including penalized logistic regression, neural networks, and gradient boosting methods. Across multiple clinically relevant endpoints, ML models demonstrated improved prognostic discrimination, highlighting their ability to incorporate nonlinear relationships and complex interactions. Importantly, demographic and comorbidity-related variables (e.g., age, diabetes, chronic kidney disease) emerged as key predictors of long-term outcomes, underscoring the importance of integrating both clinical and anatomical information into modern prognostic tools. These ML-derived prognostic findings align with long-term outcome patterns observed in large contemporary CTO registries, including the Canadian Multicenter CTO Registry [41]. This finding underscores the ability of ML to synthesize multidimensional data beyond procedural characteristics.

Explainable AI in Prognostic Models

The “black-box” nature of AI predictions remains a barrier to clinical adoption. To address this, Kim et al. applied SHAP (Shapley Additive Explanations) [40] to their models, enabling visualization of feature contributions to individual patient predictions. Higher age, renal dysfunction, and diabetes were associated with increased predicted risk, while preserved left ventricular ejection fraction and absence of major comorbidities were linked to lower predicted risk. Such interpretability tools are essential for building clinician trust and facilitating shared decision-making. Other frameworks such as LIME and counterfactual modeling have been explored in cardiovascular AI but remain underutilized in CTO-specific prognostic applications.

Integration of Patient-Reported Outcomes

An emerging area of AI prognostic modeling in CTO patients is the integration of patient-reported outcome measures (PROMs). The Seattle Angina Questionnaire (SAQ), which quantifies angina frequency, physical limitation, treatment satisfaction, and quality of life, is highly relevant in CTO populations [42]. In the EuroCTO trial [9], SAQ domains correlated with procedural success and symptom relief, yet these outcomes were not integrated into long-term prognostic models. AI could enable the integration of SAQ domains with clinical, imaging, and procedural variables, supporting more holistic, patient-centered prognostic assessment. Additional PROMs such as EQ-5D or disease-specific instruments may further enrich multimodal models.

Incorporating Imaging and Functional Data

Beyond PROMs, prognostic models can leverage imaging-derived features such as baseline LVEF, ischemic burden, and plaque morphology. Advances in echocardiography, cardiac MRI, and intravascular imaging (IVUS, OCT) provide high-dimensional inputs well suited for ML analysis. For instance, MRI-detected scar burden or IVUS-based calcium scoring—parameters linked with adverse clinical outcomes—could refine prognostic predictions when combined with demographic and comorbidity profiles. Such multimodal fusion is a promising direction for individualized risk stratification in CTO patients.

CTO vs. Non-CTO Prognostic Models

In non-CTO PCI populations, several AI models have already demonstrated strong prognostic modeling across a broad spectrum of outcomes [24]. However, CTO patients present unique clinical and anatomical complexity: older age, higher prevalence of diabetes and chronic kidney disease, greater ischemic burden, and more variable procedural success rates. These characteristics limit the performance of conventional regression-based approaches and underscore the potential incremental value of ML in CTO prognostication.

Federated Learning and Big Data Collaboration

The need for robust prognostic models underscores the importance of large, diverse datasets. Federated learning [43] has emerged as a promising strategy, enabling model training across multiple centers without centralizing patient-level data. This approach preserves privacy and addresses regulatory constraints such as GDPR, while still producing models that are more generalizable. Early applications of federated AI in arrhythmia detection and heart failure prediction suggest feasibility, and translation to CTO registries such as EuroCTO, PROGRESS-CTO, and OPEN-CTO could accelerate the development of validated prognostic models.

Implementation, Ethics, and Regulatory Perspectives

Implementation of prognostic AI in routine CTO PCI practice will require integration into clinical workflows. Ideally, clinicians would receive individualized risk scores at the bedside or in the catheterization laboratory, incorporating survival estimates, MACCE risk, and expected quality-of-life outcomes. Ethical considerations include mitigating algorithmic bias, ensuring equitable representation of diverse populations, and clarifying accountability when AI-influenced decisions lead to adverse events. Regulators such as the FDA and EMA are increasingly focusing on interpretability and safety in approving AI-based prognostic tools, making explainable AI frameworks essential for clinical adoption [24,44,45].

**Figure 4:**
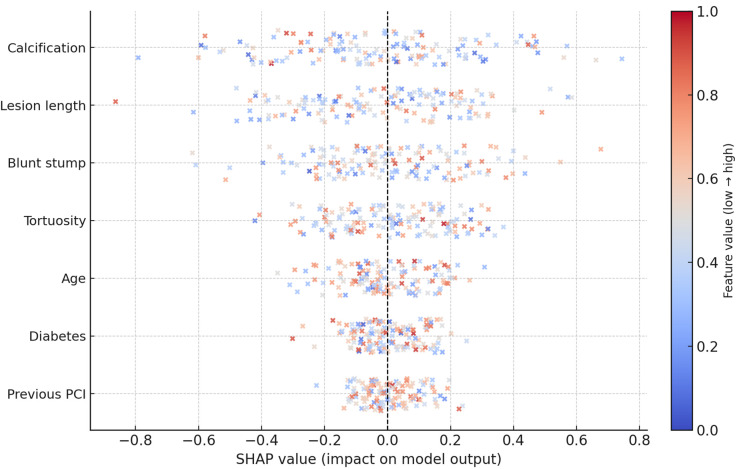
SHAP summary (schematic) for predictors of CTO PCI success.

AI-based prognostic modeling represents a significant advance in CTO management. Compared with conventional statistical approaches, ML models (particularly CatBoost and neural networks) achieve superior accuracy for predicting 5-year mortality and MACCE. They highlight the prognostic importance of comorbidities and functional status, while explainable AI enhances transparency. Integration of PROMs, imaging, and functional data can further refine prognostication, offering a holistic and patient-centered approach. Federated learning and international collaboration hold the key to developing robust, generalizable models. Figure 4 provides a schematic illustration of how multimodal data (clinical, imaging, procedural, and PROMs) can be integrated into an AI prognostic model for CTO PCI. This workflow is conceptual and intended to summarize published approaches rather than to represent an original predictive algorithm.

This schematic representation demonstrates how clinical, anatomical, and functional variables may influence individual predictions in ML models. Features such as age, kidney function, diabetes, lesion characteristics, and left ventricular ejection fraction can differentially affect predicted risk. The illustration is conceptual and does not derive from a specific dataset.

### 3.4. AI in CTO PCI vs. Non-CTO PCI

The clinical and technical challenges of percutaneous coronary intervention (PCI) vary profoundly depending on whether the target lesion is a chronic total occlusion (CTO) or a non-CTO stenosis. Artificial intelligence (AI) has found applications across both scenarios, yet the magnitude and type of added value differ meaningfully. In non-CTO PCI, where diagnostic clarity and procedural success rates are already high, AI primarily contributes to workflow optimization and automation. By contrast, CTO PCI, characterized by lesion ambiguity, higher complication risks, and more heterogeneous operator outcomes, provides a setting in which AI may offer broader and potentially more clinically relevant support.

Diagnostic Imaging: routine support vs. strategic necessity

AI-driven imaging tools are increasingly applied in both non-CTO and CTO settings. In non-CTO lesions, AI primarily enhances efficiency and reproducibility. For example, deep learning models can automatically segment coronary arteries on CCTA or angiography, detect stenotic plaques, and quantify vessel lumen dimensions with accuracy comparable to expert readers [27,28]. Intravascular imaging modalities such as IVUS and OCT also benefit from machine learning algorithms capable of automated calcium scoring, lumen detection, and plaque characterization [37,38]. These advances reduce inter-observer variability and speed up workflows, but for most non-CTO lesions, lesion morphology is straightforward, and experienced operators can already interpret imaging reliably. Thus, AI serves mainly as a time-saving adjunct in non-CTO PCI.

The situation differs dramatically in CTO PCI. CTO lesions are often characterized by ambiguous proximal caps, diffuse calcification, bridging collaterals, and lesion lengths exceeding 20 mm. These features make imaging interpretation not merely confirmatory but strategically decisive. Distinguishing subtotal occlusions (STO) from true CTOs is one of the areas where AI has already demonstrated superiority: radiomics and deep learning applied to CCTA datasets significantly improve discrimination between borderline lesions [39]. Moreover, AI algorithms can quantify calcium burden, identify optimal entry sites for wire crossing, and integrate three-dimensional reconstructions to suggest antegrade versus retrograde strategies. These capabilities go beyond efficiency, constituting a strategic necessity for procedural planning in CTO PCI.

Procedural Success Prediction: ceiling effect vs. unmet need

Procedural success in non-CTO PCI exceeds 95% in contemporary registries, particularly in high-volume centers. This creates a “ceiling effect” where predictive models—whether statistical or AI-based—have limited opportunity to demonstrate incremental accuracy. Risk prediction in non-CTO PCI often focuses on complications (e.g., contrast nephropathy, periprocedural MI), but for technical success itself, the role of AI is relatively minor.

By contrast, CTO PCI remains one of the last frontiers of variability in interventional cardiology. Success rates differ widely across institutions: 60–70% in less experienced programs versus >90% in dedicated CTO centers [7,16]. Traditional angiographic scores such as J-CTO and PROGRESS-CTO [5,6,21] provide some predictive value but are limited by their reliance on a small number of anatomical variables and lack of incorporation of operator and center-related factors. Machine learning models such as XGBoost, LightGBM, and CatBoost [29,30,32] have demonstrated higher predictive accuracy in published cohorts and can incorporate procedural strategy, operator experience, and center characteristics. In this context, AI provides a complementary tool for individualized prediction of CTO PCI success, supporting case selection and patient counseling.

Prognostic Modeling: established frameworks vs. gaps in evidence

In non-CTO PCI populations, long-term prognosis has been extensively studied in large-scale randomized trials and registries. Tools such as the GRACE and TIMI risk scores, alongside Cox regression-based models, remain robust for predicting outcomes after acute coronary syndromes and stable CAD. AI models have been explored in these contexts, often achieving AUC values >0.85 for mortality prediction [24]. However, the incremental benefit of AI is less dramatic because conventional models already perform well and are well validated across diverse populations.

In CTO PCI, prognostic modeling is more complex and less standardized. CTO patients tend to be older, with higher rates of diabetes, chronic kidney disease, peripheral artery disease, and reduced left ventricular ejection fraction [3,4]. Persistent ischemia and incomplete revascularization contribute to worse outcomes compared with non-CTO populations. Conventional CAD risk scores often underestimate risk in CTO patients, failing to capture the added burden of lesion complexity and operator variability. Machine learning prognostic models, such as those by Kim et al. [31], directly address this gap by incorporating multidimensional data, including comorbidities, procedural details, and even patient-reported outcomes (PROMs). These models consistently outperform conventional approaches, offering more accurate stratification of mortality, MACCE, and quality-of-life endpoints.

The role of explainability and data integration

**Figure 5:**
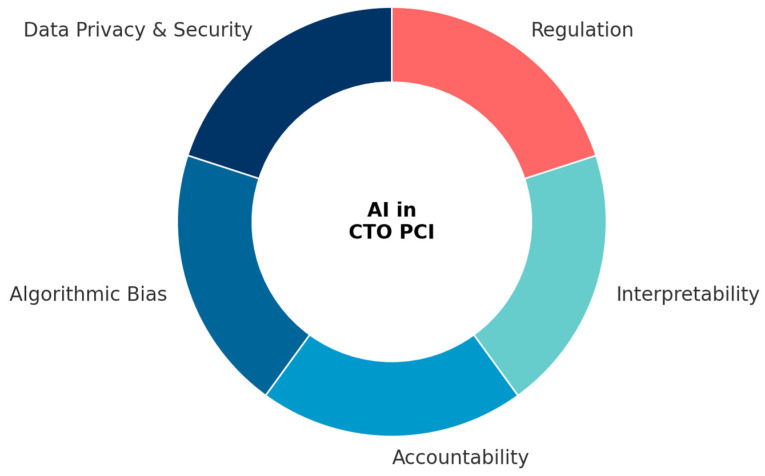
Key Implementation Challenges in AI for CTO PCI. The wheel diagram summarizes the five major domains that influence the safe and effective clinical translation of AI in chronic total occlusion percutaneous coronary intervention (CTO PCI). Data privacy and security reflect the need for compliance with GDPR and HIPAA frameworks, as well as the development of federated learning strategies to enable secure data collaboration. Algorithmic bias encompasses population-specific, vendor-dependent, and outcome definition biases, requiring diverse datasets, fairness metrics, and robust external validation. Accountability highlights unresolved questions of liability when AI contributes to clinical decision-making, emphasizing the importance of transparent frameworks for shared responsibility. Interpretability underscores the role of explainable AI (e.g., SHAP, LIME, Grad-CAM) in fostering physician trust and clinical adoption. Finally, regulation refers to evolving FDA and EMA guidelines for adaptive AI/ML software as medical devices, underscoring the necessity for international harmonization.

Another dimension of comparison is interpretability. In non-CTO PCI, the “black box” concern of AI is less acute because decisions are generally less complex. In CTO PCI, however, operator trust in AI models is paramount: decisions such as antegrade versus retrograde approach, case selection, or procedural abandonment carry major implications. Techniques such as SHAP (Figure 5) allow visualization of individual-level risk contributions, making AI not only more accurate but also more clinically transparent in CTO cases. Similarly, the integration of multimodal data—including CCTA, IVUS, OCT, baseline LVEF, and PROMs—offers far greater incremental value in CTO PCI than in non-CTO lesions, where data heterogeneity is less pronounced.

Conclusion: greater added value in CTO PCI

The comparison between CTO and non-CTO PCI highlights a key principle: the added value of AI tends to increase with the complexity and variability of the clinical scenario. In non-CTO PCI, where diagnostic clarity is high, success rates approach 100%, and validated prognostic scores already exist, AI provides mainly efficiency and standardization. By contrast, in CTO PCI, where imaging interpretation guides strategy, procedural success is variable, and prognostic tools are underdeveloped, AI contributes at every stage of care: from pre-procedural planning, through intra-procedural decision support, to long-term risk prediction. For this reason, CTO PCI may be considered an “ideal proving ground” for AI applications in interventional cardiology, with potential to redefine both operator practice and patient outcomes. To provide a concise overview of these differences, Table 2 summarizes the comparative value of AI in CTO versus non-CTO PCI across key domains of imaging, procedural success, prognosis, and interpretability.

## 4. Implementation Challenges, Bias, and Ethics

Artificial intelligence (AI) has shown great promise in improving diagnosis, procedural planning, and long-term prognostication in chronic total occlusion (CTO) percutaneous coronary intervention (PCI). However, translating these advances into everyday clinical practice requires addressing a number of significant challenges. These challenges include issues of data privacy, algorithmic bias, accountability, interpretability, and regulatory oversight. Without careful attention to these domains, AI risks remaining confined to research settings, with limited clinical impact.

### 4.1. Data Privacy and Security

The integration of multimodal data—including coronary computed tomography angiography (CCTA), intravascular ultrasound (IVUS), optical coherence tomography (OCT), and patient-reported outcome measures (PROMs)—inevitably raises questions of data privacy and patient confidentiality. Within the European Union, the General Data Protection Regulation (GDPR) imposes strict limitations on the sharing of identifiable health data, while in the United States, the Health Insurance Portability and Accountability Act (HIPAA) enforces similar standards. These legal frameworks are essential to protect patients but also limit large-scale data pooling, which is crucial for the development of robust AI algorithms.

One promising strategy to overcome these restrictions is the use of federated learning. In this framework, AI models are trained locally at multiple institutions, and only the model parameters are shared with a central aggregator, rather than the raw data. This approach enables collaborative learning without direct data centralization and has been shown to be feasible in medical imaging applications [33]. Nevertheless, federated learning also brings technical challenges, such as harmonizing imaging protocols across centers, ensuring uniform data quality, and addressing legal complexities in cross-border collaborations. Regulatory acceptance of federated learning is still in its early stages, but its potential to facilitate privacy-preserving international research in CTO PCI is considerable.

### 4.2. Algorithmic Bias

Another major challenge lies in algorithmic bias. If not properly addressed, AI models may inadvertently perpetuate or amplify existing healthcare disparities [26,34]. In CTO PCI, population bias is particularly evident: most models have been trained and validated in Asian and European cohorts [29,30,31,32], which may not reflect the demographic or clinical characteristics of patients from North America, Africa, or Latin America. Consequently, the performance of these algorithms may decline when applied in underrepresented populations.

Device or vendor bias is another concern. Algorithms developed using imaging data from a single manufacturer’s platform—whether CCTA scanners, IVUS systems, or OCT consoles—may not generalize to other devices. In addition, outcome definition bias further complicates the field, as registries often use differing endpoints for “procedural success.” Some define success as guidewire crossing, while others require restoration of TIMI 3 flow, and such variability undermines the comparability and generalizability of models.

Mitigating these biases requires a multifaceted approach. Diverse and multi-ethnic datasets should be incorporated whenever possible. The application of fairness metrics, such as equal opportunity difference and demographic parity, can provide quantitative assessments of whether AI models perform consistently across subgroups [26]. External validation in independent datasets is also essential, and federated learning offers a mechanism to ensure that training cohorts reflect broader patient populations [33].

### 4.3. Accountability and Responsibility

The question of accountability represents another unresolved barrier to clinical adoption. Current frameworks, such as those developed by the U.S. Food and Drug Administration (FDA), classify AI algorithms as “Software as a Medical Device (SaMD),” meaning they are considered assistive tools rather than autonomous decision-makers [35]. As such, responsibility for treatment decisions remains with the physician. However, as AI systems become more advanced and are integrated into real-time procedural support, the boundaries of accountability may become blurred.

A complication arises when AI recommendations contribute to procedural outcomes, especially in high-risk interventions such as CTO PCI. In such cases, should liability rest with the interventionalist, the hospital, the algorithm developer, or the regulatory body that approved the tool? Legal scholars suggest that contractual agreements, explicit institutional policies, and revised informed consent procedures may help clarify these responsibilities. Ultimately, accountability must remain transparent and shared, ensuring that patient trust in both physicians and AI tools is preserved.

### 4.4. Interpretability and Explainability

The interpretability of AI models is perhaps one of the most important practical considerations for clinical integration. Many AI models function as “black boxes,” producing predictions without clear rationale. For clinicians, especially in a field as complex as CTO PCI, trust in an algorithm requires understanding not only what the prediction is, but also why it was made. Explainable AI (XAI) techniques are therefore indispensable.

Several methods are particularly relevant. Shapley Additive Explanations (SHAP) provide insight into the contribution of individual features to both global model performance and patient-specific predictions [40]. Local Interpretable Model-Agnostic Explanations (LIME) create simplified surrogate models that can approximate the behavior of more complex algorithms. In imaging, Gradient-weighted Class Activation Mapping (Grad-CAM) highlights the specific regions of a scan that drive AI decisions, offering a visual explanation that aligns with physician intuition. These methods can help clinicians validate algorithmic predictions against their own expertise, thereby improving trust and adoption.

### 4.5. Regulatory Frameworks

Finally, regulatory frameworks are evolving but remain incomplete. The FDA has issued a proposed framework for adaptive AI and machine learning-based models [35]. Unlike static algorithms, adaptive models update continuously as new data are introduced, raising unique challenges for validation and safety oversight. The FDA recommends pre-specified update protocols, continuous post-market surveillance, and real-world performance monitoring.

The European Medicines Agency (EMA) has also recognized the need for rigorous standards, publishing guidelines on the clinical evaluation of medical device software [36]. These guidelines emphasize external validation, reproducibility, and compliance with GDPR. However, specific requirements for AI applications in interventional cardiology, and CTO PCI in particular, are not yet fully established. International harmonization will be essential to ensure that AI tools developed in one jurisdiction can be safely adopted in another.

The implementation of AI in CTO PCI is hindered by several interrelated challenges. Data privacy regulations such as GDPR and HIPAA restrict unrestricted data sharing, though federated learning offers a pathway to privacy-preserving collaboration. Algorithmic bias, including population, vendor, and outcome definition biases, threatens the generalizability and equity of AI models, but can be mitigated by fairness metrics, diverse training cohorts, and external validation. Accountability remains a debated issue, particularly as AI evolves from assistive to potentially semi-autonomous roles. Interpretability is essential for clinical trust, and XAI techniques such as SHAP, LIME, and Grad-CAM represent important advances. Finally, regulatory frameworks from the FDA and EMA are progressing but require greater international coordination.

By proactively addressing these challenges, AI can transition from retrospective promise to prospective reality, providing equitable, transparent, and clinically meaningful support for interventional cardiologists and their patients. These challenges are summarized schematically in Figure 5, which illustrates the five key domains—data privacy, algorithmic bias, accountability, interpretability, and regulation—that shape the safe and effective implementation of AI in CTO PCI.

## 5. Future Perspectives

Artificial intelligence (AI) in chronic total occlusion (CTO) percutaneous coronary intervention (PCI) has thus far demonstrated substantial potential in diagnostic imaging, procedural planning, and prognostic modeling. Yet, the current body of evidence remains largely retrospective, single-center, and exploratory. To translate AI from proof-of-concept into widespread clinical adoption, future research must address unmet needs while embracing technological innovations that are likely to reshape the interventional landscape. Several forward-looking directions can be identified, ranging from real-time guidance and robotics integration to federated learning, multimodality AI, training applications, and large-scale registry collaboration.

### 5.1. Real-Time AI Guidance in the Catheterization Laboratory

Perhaps the most transformative vision for AI in CTO PCI is the integration of real-time guidance directly into catheterization laboratory workflows. Most current AI applications are retrospective, predicting procedural success or long-term prognosis before the intervention. However, dynamic intra-procedural support could offer immediate benefits. For instance, AI algorithms trained on angiographic and intravascular imaging datasets could provide live lesion assessment, automated segmentation of calcified plaques, and dynamic visualization of the most probable wire entry path [46,47].

Preliminary data from non-CTO PCI indicate that real-time AI can accurately quantify vessel diameter, lesion length, and plaque burden during angiography, thereby reducing inter-operator variability [48]. Extending such applications to CTO PCI could shorten procedure times, reduce contrast use, and minimize radiation exposure. Importantly, prospective multicenter studies will be needed to validate whether real-time AI guidance leads not only to greater efficiency but also to improved clinical outcomes. Integration into existing catheterization hardware and imaging platforms will require close collaboration between industry, engineers, and clinicians.

### 5.2. Robotics Integration and Semi-Autonomous PCI

Robotic-assisted PCI has already demonstrated benefits in reducing operator radiation exposure, improving precision in stent deployment, and enabling remote interventions [49,50]. Robotic-assisted PCI has already demonstrated benefits in reducing operator radiation exposure, improving precision in stent deployment, and enabling remote interventions [49,50]. Previous reviews have further summarized the technical advantages and emerging clinical applications of robotic coronary angioplasty [51]. The next step lies in combining robotics with AI to create semi-autonomous systems. The next step lies in combining robotics with AI to create semi-autonomous systems. In this scenario, AI algorithms could analyze pre-procedural CCTA or intra-procedural IVUS and OCT to suggest optimal wire trajectories or to control robotic guidewire navigation. The interventionalist would remain in command, but AI-driven robotics could minimize wire trauma, avoid subintimal misdirection, and standardize complex techniques such as retrograde crossing or dissection–reentry [52].

Beyond procedural execution, AI-enabled robotics could also play a role in training and simulation. By embedding AI guidance into robotic simulators, less experienced operators could rehearse CTO procedures in a safe, controlled environment, gradually building confidence before tackling real-world cases. Ultimately, such systems may democratize access to CTO PCI, narrowing the gap between high-volume expert centers and smaller institutions.

### 5.3. Federated Learning and Collaborative AI Development

One of the most critical bottlenecks in AI for CTO PCI is data availability. Large-scale model development requires diverse, high-quality datasets that span geographies, device vendors, and patient populations. Yet, GDPR in Europe and HIPAA in the United States impose strict limitations on patient-level data sharing. Federated learning (FL) represents a paradigm-shifting solution to this problem [33].

In federated learning, AI models are trained locally at participating institutions, and only model parameters—not raw data—are transmitted to a central aggregator. This approach preserves patient privacy while enabling collaborative development of robust, generalizable algorithms. In the context of CTO PCI, FL could allow multinational centers to jointly develop models for CCTA-based lesion detection, IVUS segmentation, or outcome prediction, without ever exchanging sensitive patient data.

The advantages extend beyond privacy. By incorporating diverse imaging protocols, clinical practices, and patient demographics, FL can reduce algorithmic bias and improve model robustness [43]. Pilot studies in arrhythmia detection and medical imaging have already demonstrated feasibility [33], but application to CTO registries remains limited. Future initiatives such as EuroCTO, PROGRESS-CTO, and OPEN-CTO could serve as federated platforms, catalyzing international collaboration while fully respecting legal frameworks.

### 5.4. Multimodality AI: Integrating Imaging, Clinical, and Patient-Reported Data

Another key frontier is the development of multimodality AI systems that fuse diverse datasets into unified prognostic and procedural platforms. Current models often focus on a single modality—such as angiographic scores, CCTA features, or comorbidity profiles. Yet CTO PCI outcomes are inherently multifactorial, shaped by anatomical complexity, operator expertise, and patient comorbidities.

Future AI models should aim to integrate pre-procedural CCTA with intra-procedural IVUS and OCT, as well as with angiographic data, functional parameters such as left ventricular ejection fraction, and patient-reported outcomes such as Seattle Angina Questionnaire (SAQ) scores [42]. Such multimodality systems could simultaneously optimize procedural planning and provide patient-centered prognostic insights. For example, an AI tool might predict not only the likelihood of technical success but also the expected improvement in angina frequency and quality of life, enabling more individualized shared decision-making.

A major challenge in this domain will be data harmonization. Different imaging modalities produce datasets with varying resolutions, formats, and labeling standards. Advanced techniques such as deep feature fusion and graph neural networks may facilitate cross-modality integration, but rigorous validation will be required before clinical use.

### 5.5. AI in Education, Simulation, and Training

The complexity of CTO PCI poses steep learning curves, even for experienced interventionalists. AI holds promise in accelerating operator training through immersive educational platforms. Virtual reality (VR) and augmented reality (AR) simulators powered by AI can provide realistic, patient-specific rehearsal environments [52]. Such systems could incorporate anonymized CCTA or angiographic data from actual patients, allowing interventionalists to “practice” the procedure before entering the catheterization laboratory.

Furthermore, AI-based feedback systems can objectively assess technical performance, highlighting errors such as suboptimal wire positioning or excessive fluoroscopy time. This may accelerate skill acquisition and promote standardized training across institutions. In the long run, AI-enabled training could reduce variability in procedural outcomes, improve patient safety, and expand the pool of operators capable of performing CTO PCI successfully.

### 5.6. Big Data, Registries, and International Collaboration

Finally, the future of AI in CTO PCI will be closely linked to the expansion and integration of large-scale registries. Initiatives such as EuroCTO, PROGRESS-CTO, and OPEN-CTO already provide invaluable clinical and procedural data, but their potential is far from fully realized [53]. By incorporating imaging datasets, long-term follow-up, and patient-reported outcomes, these registries could evolve into comprehensive platforms for AI development and validation.

Big data approaches offer opportunities not only for technical success prediction but also for personalized prognostication, secondary prevention strategies, and cost-effectiveness analysis. Importantly, international collaboration will be crucial to harmonize endpoint definitions, ensure data standardization, and reduce duplication of effort [43]. As federated learning matures, it may allow these registries to function as global AI development hubs without requiring central data pooling.

Summary

Future applications of AI in CTO PCI will extend far beyond retrospective risk prediction. Real-time intra-procedural guidance promises to enhance safety, efficiency, and outcomes in the catheterization laboratory. Robotics integration may enable semi-autonomous PCI, reduce operator risk, and democratize access to advanced CTO interventions. Federated learning represents a breakthrough strategy to overcome data privacy barriers, enabling international collaboration without compromising patient confidentiality. Multimodality AI systems will integrate anatomical, functional, and patient-reported data, providing holistic, patient-centered predictions. Educational applications, including VR and AI-enabled training, may shorten learning curves and standardize procedural performance. Finally, large-scale registries will serve as the backbone of AI development, validation, and clinical implementation.

**Figure 6:**
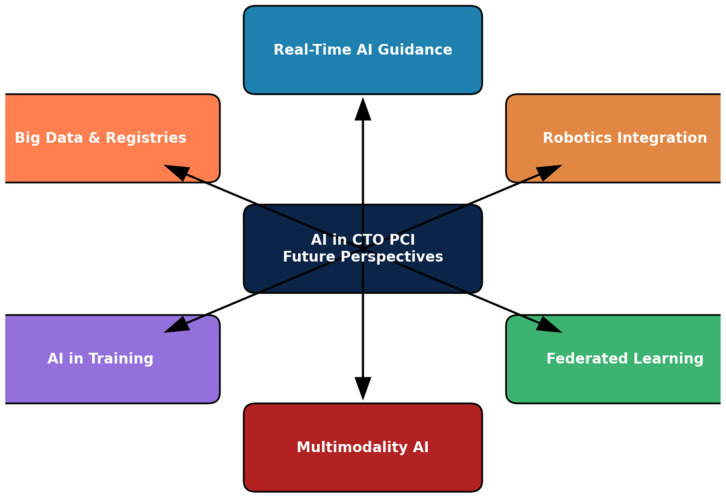
Future Perspectives of AI in CTO CPI.

Together, these perspectives underscore a future where AI is not a peripheral adjunct but a central partner in CTO PCI, enhancing both procedural precision and long-term patient care. A schematic representation of the main future perspectives of AI in CTO PCI is provided in Figure 6, illustrating how real-time decision support, robotics, federated learning, multimodality integration, educational tools, and big data registries converge toward advancing interventional cardiology.

## 6. Discussion

Artificial intelligence (AI) in chronic total occlusion (CTO) percutaneous coronary intervention (PCI) has moved from theoretical promise to tangible applications across diagnostic imaging, procedural success prediction, and long-term prognostication. The evidence synthesized in this review highlights both the opportunities and challenges associated with AI adoption in interventional cardiology.

Summary of Evidence

In diagnostic imaging, AI-driven models have consistently demonstrated accuracy comparable to expert interpretation while reducing time and variability. Deep learning applied to coronary CT angiography (CCTA) has shown high diagnostic accuracy [28], while intravascular ultrasound (IVUS) and optical coherence tomography (OCT) segmentation demonstrated excellent agreement with expert analysis [37,38]. These advances improve reproducibility and efficiency, yet CTO-specific validation remains limited.

In procedural planning, conventional angiographic scores such as J-CTO and PROGRESS-CTO have shown only modest discriminatory ability [5,6,21]. Machine learning models, including XGBoost, LightGBM, and CatBoost, have consistently surpassed these scores, achieving superior predictive performance across large multicenter datasets [29,30,32]. Importantly, some models incorporated operator experience and institutional volume, addressing a key limitation of anatomy-only risk stratification.

In long-term prognostic modeling, AI has shown equal or greater promise. Kim et al. reported that machine learning approaches outperformed regression-based methods for predicting long-term mortality [31]. Predictive performance was strongest for mortality but more modest for repeat revascularization, reflecting the complexity and variability of patient and procedural factors. Notably, comorbidities (diabetes, chronic kidney disease) and functional variables (LVEF) proved more predictive than procedural strategy, highlighting AI’s ability to integrate multidimensional patient data.

Strengths and Advantages

AI offers clear advantages: (1) ability to integrate multimodal, high-dimensional data (CCTA, IVUS, OCT, clinical variables, PROMs); (2) reproducibility and reduction of inter-observer variability; (3) dynamic adaptability, as algorithms can evolve with new datasets; and (4) potential for real-time applications, enabling intra-procedural decision support in the catheterization laboratory.

Weaknesses and Limitations

Current limitations temper these benefits. Most studies are retrospective, single-center, and small in scale, limiting generalizability. External validation remains rare, and many models lose discriminatory accuracy when tested outside the derivation cohort. Algorithmic bias is a growing concern: population bias (studies derived predominantly from Asian or European cohorts), vendor bias (platform-specific imaging data), and outcome definition bias all threaten generalizability. Without fairness metrics and diverse datasets, such biases risk perpetuating inequities. Furthermore, the “black-box” nature of many models undermines clinical trust, despite emerging explainability frameworks such as SHAP and LIME. Finally, workflow integration remains underdeveloped, with few models deployed in real-time settings.

Research Gaps and Opportunities

Future work must prioritize prospective, multicenter validation to confirm reproducibility across diverse populations. Harmonization of endpoints—technical success, MACCE, QoL—will improve comparability across studies. Federated learning offers a promising approach to overcome privacy barriers imposed by GDPR and HIPAA, enabling model training across international registries without centralizing patient data. Equally important is the integration of patient-reported outcomes, such as the Seattle Angina Questionnaire, into predictive frameworks. Educational applications, including AI-driven simulators and patient-specific procedural rehearsal, could accelerate operator training.

Critical Perspective

AI should be viewed as a powerful adjunct, not a replacement for clinical judgment. The interventionalist remains the final decision-maker, with AI functioning as a “co-pilot” to augment decision-making. Risks include overreliance, complacency, and unclear accountability in adverse outcomes. Regulatory bodies must define clear frameworks to ensure clinician responsibility and patient safety.

Future Linkage

The trajectory of AI in CTO PCI aligns with future perspectives: real-time decision support, robotics integration, multimodal fusion, and federated learning. With international collaboration and regulatory safeguards, AI can evolve from retrospective analysis to real-world application, ultimately enhancing both procedural outcomes and long-term patient-centered care.

AI outperforms conventional risk scores in CTO PCI

Machine learning consistently demonstrates higher predictive accuracy than angiographic scores, providing incremental value in procedural success and long-term prognosis.

- Multimodality is central to AI’s added value. By integrating coronary imaging (CCTA, IVUS, OCT), clinical comorbidities, procedural characteristics, and patient-reported outcomes, AI creates holistic models that extend beyond technical success to long-term quality of life.
- Federated learning will drive the next generation of models. Collaborative AI development across registries such as EuroCTO, PROGRESS-CTO, and OPEN-CTO is feasible without centralizing patient-level data. This approach improves generalizability while maintaining compliance with GDPR and HIPAA.
- Clinical translation requires prospective validation. Most current evidence is retrospective and single-center. Large, prospective, multicenter trials with harmonized endpoints are essential for routine clinical integration.
- Bias mitigation and interpretability are prerequisites for adoption. Algorithmic bias—population, vendor, and outcome-related—must be actively addressed. Explainable AI frameworks (SHAP, LIME) are crucial to improve transparency, foster clinician trust, and satisfy regulatory standards.

## Figures and Tables

**Table 1:** Comparative performance of traditional angiographic scores and ML models for prediction of procedural success in CTO PCI.

Ref.	Study/Year	Population	Model(s) Tested	Comparator(s)	Endpoint	AUC (Qualitative)	Validation/Notes
[5]	Morino et al., 2011	Multicenter CTO registry	J-CTO score	—	Guidewire crossing ≤ 30 min	Moderate discrimination	Internal validation; anatomy-based variables only
[6]	Christopoulos et al., 2016	PROGRESS-CTO registry	PROGRESS-CTO	—	Technical success	Modest predictive capacity	Anatomy-focused; limited external validation
[15]	Maeremans et al., 2016	RECHARGE registry	RECHARGE score	—	Technical success	Moderate discrimination	Procedural + anatomical parameters
[21]	Szijgyarto et al., 2019	EuroCTO CASTLE cohort	CASTLE score	—	Technical failure prediction	Moderate performance	External validation available; anatomy-driven
[29]	Nakachi et al., 2023	8760 CTO PCI cases (Japan)	XGBoost	J-CTO, CL, CASTLE	Technical success	Higher discriminatory performance vs. angiographic scores	Cross-validation; clinical + anatomical variables
[30]	Nakachi et al., 2023	Multicenter CTO cohort	XGBoost, other ML models	J-CTO	Guidewire crossing success	ML models outperformed conventional scores	Train/test split; gradient boosting highest performance

The table provides a qualitative summary of key studies evaluating traditional angiographic scoring systems (J-CTO, PROGRESS-CTO, RECHARGE, CASTLE) and machine-learning–based models (primarily XGBoost) for predicting procedural success in CTO PCI. For each study, the table outlines the type of model, the comparator, the primary endpoint, and a qualitative assessment of predictive performance. Across published studies, ML approaches generally demonstrate higher discriminatory performance than conventional scores, especially when incorporating both anatomical and clinical variables.

**Table 2:** Comparative value of AI in CTO vs. Non-CTO PCI.

Domain	Non-CTO PCI	CTO PCI	Incremental Value of AI
Diagnostic imaging	Automates lumen segmentation, plaque quantification, improves efficiency and reproducibility [27,28,37]	Guides strategic planning (CTO vs. STO differentiation, calcium burden, entry site identification) [29,39]	High in CTO (strategic necessity) vs. Moderate in non-CTO (workflow efficiency)
Procedural success prediction	Limited utility due to >95% baseline success (‘ceiling effect’)	Success rates 60–90%; AI outperforms J-CTO/PROGRESS-CTO by integrating clinical + operator + center-level factors [29,30,32]	Critical unmet need in CTO; minor role in non-CTO
Prognostic modeling	Conventional risk scores (GRACE, TIMI) robust and validated; AI adds incremental accuracy [24]	Conventional models underperform; AI integrates comorbidities, imaging, PROMs → improved prediction of mortality, MACCE, QoL [31,42]	Substantial in CTO; incremental in non-CTO
Explainability & interpretability	Lower clinical impact; outcomes less variable	SHAP, LIME crucial for operator trust and patient counseling [40]	Essential in CTO; secondary in non-CTO
Overall clinical impact	Efficiency, standardization, automation	Multilevel value: planning, success prediction, prognosis	AI provides greatest added value in CTO PCI

## Data Availability

Not applicable.

## Abbreviations

The following abbreviations are used in this manuscript:

AI	Artificial Intelligence
AUC	Area Under the Curve
AR	Augmented Reality
CABG	Coronary Artery Bypass Grafting
CAD	Coronary Artery Disease
CASTLE	CASTLE (CTO technical failure-risk score)
CatBoost	Categorical Boosting
CCTA	Coronary Computed Tomography Angiography
CNN	Convolutional Neural Network
CTO	Chronic Total Occlusion
DECISION-CTO	DECISION-CTO trial
DL	Deep Learning
EQ-5D	EuroQol 5-Dimension questionnaire
EXPLORE	EXPLORE trial
EuroCTO	European Chronic Total Occlusion trial
FDA	United States Food and Drug Administration
FFR-CT	Fractional Flow Reserve derived from CT
FL	Federated Learning
GDPR	General Data Protection Regulation
Grad-CAM	Gradient-weighted Class Activation Mapping
HIPAA	Health Insurance Portability and Accountability Act
IVUS	Intravascular Ultrasound
J-CTO	Japanese Chronic Total Occlusion score
LightGBM	Light Gradient Boosting Machine
LIME	Local Interpretable Model-Agnostic Explanations
LR	Logistic Regression
LVEF	Left Ventricular Ejection Fraction
MACE	Major Adverse Cardiac and Cerebrovascular Events
ML	Machine Learning
MRI	Magnetic Resonance Imaging
OCT	Optical Coherence Tomography
OMT	Optimal Medical Therapy
OPEN-CTO	Open Chronic Total Occlusion registry
PCI	Percutaneous Coronary Intervention
PRISMA	Preferred Reporting Items for Systematic Reviews and Meta-Analyses
PROGRESS-CTO	Prospective Global Registry for CTO Interventions
PROMs	Patient-Reported Outcome Measures
QoL	Quality of Life
RECHARGE	RECHARGE registry
RF	Random Forest
SAQ	Seattle Angina Questionnaire
SCAI	Society for Cardiovascular Angiography and Interventions
SHAP	Shapley Additive Explanations
STO	Subtotal Occlusion
SVM	Support Vector Machine
TIMI	Thrombolysis in Myocardial Infarction
TVR	Target Vessel Revascularization
VR	Virtual Reality
XAI	Explainable Artificial Intelligence
XGBoost	Extreme Gradient Boosting
